# H3K27me3 expression and methylation status in histological variants of malignant peripheral nerve sheath tumours

**DOI:** 10.1002/path.5507

**Published:** 2020-09-01

**Authors:** Iben Lyskjær, Daniel Lindsay, Roberto Tirabosco, Christopher D Steele, Patrick Lombard, Anna‐Christina Strobl, Ana M Rocha, Christopher Davies, Hongtao Ye, Elise Bekers, Julia Ingruber, Matt Lechner, Fernanda Amary, Nischalan Pillay, Adrienne M Flanagan

**Affiliations:** ^1^ Research Department of Pathology University College London London UK; ^2^ Department of Histopathology Royal National Orthopaedic Hospital Stanmore UK; ^3^ Department of Pathology Netherlands Cancer Institute Amsterdam The Netherlands; ^4^ Department of Otorhinolaryngology Medical University of Innsbruck Innsbruck Austria; ^5^ UCL Cancer Institute, University College London London UK

**Keywords:** malignant peripheral nerve sheath tumours, H3K27me3, whole‐exome sequencing, whole‐genome sequencing, DNA methylation, sarcoma, genome doubling, neurofibromatosis type 1; RNA‐sequencing

## Abstract

Diagnosing MPNST can be challenging, but genetic alterations recently identified in polycomb repressive complex 2 (PRC2) core component genes, *EED* and *SUZ12*, resulting in global loss of the histone 3 lysine 27 trimethylation (H3K27me3) epigenetic mark, represent drivers of malignancy and a valuable diagnostic tool. However, the reported loss of H3K27me3 expression ranges from 35% to 84%. We show that advances in molecular pathology now allow many MPNST mimics to be classified confidently. We confirm that MPNSTs harbouring mutations in PRC2 core components are associated with loss of H3K27me3 expression; whole‐genome doubling was detected in 68%, and *SSTR2* was amplified in 32% of MPNSTs. We demonstrate that loss of H3K27me3 expression occurs overall in 38% of MPNSTs, but is lost in 76% of histologically classical cases, whereas loss was detected in only 23% cases with heterologous elements and 14% where the diagnosis could not be provided on morphology alone. H3K27me3 loss is rarely seen in other high‐grade sarcomas and was not found to be associated with an inferior outcome in MPNST. We show that DNA methylation profiling distinguishes MPNST from its histological mimics, was unrelated to anatomical site, and formed two main clusters, MeGroups 4 and 5. MeGroup 4 represents classical MPNSTs lacking H3K27me3 expression in the majority of cases, whereas MeGroup 5 comprises MPNSTs exhibiting non‐classical histology and expressing H3K27me3 and cluster with undifferentiated sarcomas. The two MeGroups are distinguished by differentially methylated PRC2‐associated genes, the majority of which are hypermethylated in the promoter regions in MeGroup 4, indicating that the PRC2 target genes are not expressed in these tumours. The methylation profiles of MPNSTs with retention of H3K27me3 in MeGroups 4 and 5 are independent of mutations in PRC2 core components and the driver(s) in these groups remain to be identified. Our results open new avenues of investigation. © 2020 The Authors. *The Journal of Pathology* published by John Wiley & Sons, Ltd. on behalf of The Pathological Society of Great Britain and Ireland.

## Introduction

Malignant peripheral nerve sheath tumours (MPNSTs) are characterised by nerve sheath differentiation and account for roughly 5% of all sarcoma subtypes [1]. Approximately 45% present in the context of neurofibromatosis type‐1 (NF‐1), a germline tumour predisposition syndrome (incidence ~1/3,500) caused by constitutional mutations in the tumour suppressor gene *neurofibromin 1* (*NF1*); 45% of cases occur as sporadic tumours and 10% are radiation‐associated. MPNST has an aggressive clinical course, with 50% relapsing and an overall average 5‐year survival rate of 43% [2]. The classical variant of MPNST shows features of a monomorphic spindle cell sarcoma with alternating hypercellular and hypocellular areas and geographic tumour necrosis. Heterologous sarcomatous differentiation may also be seen, the most common being rhabdomyoblastic differentiation (Triton tumour) [1]. However, the histology is not always typical and MPNSTs may exhibit significant pleomorphism [3]. The differential diagnoses include, amongst others, spindle cell sarcoma not otherwise specified (NOS), synovial sarcoma, dermatofibrosarcoma protuberans, spindle cell rhabdomyosarcoma (SC‐RMS), and melanoma [1].

Detection of somatic *NF1* genetic alterations has been reported in sporadic and radiation‐associated MPNST, but with variable detection rates ranging from 44% to 100% [4, 5, 6]. *NF1* is a regulator of Ras protein activity and loss of function (LOF) of *NF1* results in hyperactivation of the Ras pathway and excessive proliferation. MPNST is characterised by LOF of additional tumour suppressor loci, including p16^INK4A^ (*CDKN2A*) and *TP53*; however, these alterations are also seen in atypical neurofibroma, the presumed precursor of MPNST. In contrast, promotion of malignancy has been reported to be associated with recurrent somatic LOF alterations in genes encoding the polycomb repressive complex 2 (PRC2) core components, *Embryonic Ectoderm Development* (*EED*) and *Suppressor of Zeste 12* (*SUZ12*). These have been identified in 23–70% of NF‐1‐associated, 36–92% of sporadic, and 90% of radiation‐associated MPNSTs [4, 5, 7]. Subsequently, loss of H3K27me3 expression has been shown to correlate strongly with high‐grade MPNST harbouring mutations in *EED* and *SUZ12*, consistent with the knowledge that the PRC2 complex trimethylates H3K27 [3]. However, in follow‐up studies, the reported loss of H3K27me3 immunoreactivity has been variable, ranging from 34% [8] to 84% of cases [3, 4, 9, 10, 11].

Röhrich *et al* reported that high‐grade nerve sheath tumours clustered on the basis of their DNA methylation status, data that complemented the known role of the PRC2 complex in methylating H3K27. They identified two distinct clusters, the larger associated with loss of H3K27me3 immunoreactivity and the smaller group, sited in a paraspinal location, which retained expression of this protein [12]. Moreover, they and others demonstrated that loss of H3K27me3 immunoreactivity was valuable for distinguishing neurofibroma and low‐grade MPNST from high‐grade disease [3, 4, 8, 9, 13, 14]. In this study, we investigate further the genomic and epigenomic landscape of MPNST and H3K27me3 expression in the context of morphologically distinct groups of MPNST.

## Materials and methods

Ethical approval was obtained from the Cambridgeshire 2 Research Ethics Service (09/H0308/165).

### Sample selection and identification of clinical information

All cases were retrieved from the Royal National Orthopaedic Hospital (RNOH) pathology archives between 1 January 2003 and 21 December 2015 (supplementary material, Table S1). A collection of undifferentiated pleomorphic sarcomas (UPSs), subjected to whole‐genome sequencing (WGS) in a previous study, was also studied [15]. A spinal/paraspinal tumour was defined on imaging as arising from or involving a large spinal nerve route (supplementary material, Supplementary materials and methods).

Cases (totalling 176) where a diagnosis of MPNST had been considered in the differential diagnosis with a variable degree of confidence were selected for the study (by RT, DL, FA, and AMF). Additional IHC and molecular tests were performed where required to provide a more definitive diagnosis. Examples of the original reports of such cases included ‘a spindle cell sarcoma NOS with features raising the possibility of a MPNST’ or ‘the diagnosis includes an MPNST with rhabdomyoblastic differentiation and an SC‐RMS’. Following review, the diagnosis of the latter would have been confirmed by detection of the MYO‐D1 single‐nucleotide variant (SNV) p.L122R. Following this review, we classified the cases into ten histopathological groups (Table 1): 124 were considered to represent nerve sheath tumours in Histopathology Groups (HP Groups) 0, 1A, 1B, and 2 (Table 2). Diagnoses were made according to WHO and Trojani guidelines [16] (supplementary material, Supplementary materials and methods).

**Table 1:** Histopathological groups (HP Grps).

Histopathological group (*n*)	Description
HP Grp 0 (15)	Benign nerve sheath tumours and MPNST grade 1
HP Grp 1A (46)	Classical MPNST; fascicles with monomorphic spindle cells with alternating hypercellular and hypocellular areas and geographic tumour necrosis without heterologous elements
HP Grp 1B (31)	Classical MPNST; spindle cell sarcoma with heterologous element, e.g. rhabdomyoblastic, osteosarcoma; also, MPNST with a variable degree of cellular pleomorphism but in which a classical low‐grade MPNST component was identified
HP Grp 2 (41)	MPNST in the absence of classical histological morphology, e.g. tumours exhibiting scattered atypia/pleomorphism, but where the diagnosis was supported either with additional phenotypic, genomic data pointing towards MPNST or arising from a large nerve
HP Grp 3 (21)	Spindle cell sarcoma NOS (DNA sequencing not available) which does not exhibit typical MPNST morphology and without other evidence, as listed in Grp 2, to support a diagnosis of MPNST
HP Grp 4 (7)	Sarcoma with specific diagnosis based on genetic alterations including *SYT–SSX, COL1A1–PDGFB*, and others
HP Grp 5 (4)	Epithelioid MPNST requiring loss of INI‐1
HP Grp 6 (1)	Undifferentiated pleomorphic sarcoma (UPS); tumour set had undergone WGS [15]
HP Grp 7 (5)	Spindle cell/sclerosing‐RMS (SC‐RMS) with *MYOD1* mutation
HP Grp 8 (5)	Melanoma supported by publicly available genomic data [29]

**Table 2:** Clinicopathological features of 51 cases subjected to exome sequencing.

Case	Age*	Anatomical site	Site	Gender	Arising from a nerve	Clinical features of NF1	Histopathological subtype	Associated low‐grade component	Tumour grade	Primary orlocal recurrence	H3K27me3 IHC status	Radiation field
1	80	Forearm nerve‐associated	Extremity	Female	Yes	No	HP Grp 1B	NA	2	Primary	Retained	No
2	48	Tibial nerve	Extremity	Female	Yes	Yes	HP Grp 1B	Neurofibroma	3	Primary	Retained	No
3	47	Hand	Extremity	Female	No	No	HP Grp 2	NA	3	Primary	Loss	No
4	22	Buttock	Extremity	Male	No	Yes	HP Grp 2	NA	3	Primary	Loss	No
5	49	Hip	Extremity	Male	Possibly	No	HP Grp 2	NA	3	Primary	Retained	No
7	38	Buttock	Extremity	Female	No	No	HP Grp 1A	NA	3	Primary	Retained	No
8	18	Hand	Extremity	Male	No	Yes	HP Grp 1A	NA	3	Primary	Loss	No
9	56	Thigh	Extremity	Female	No	Yes	HP Grp 2	NA	3	Primary	Retained	No
10	34	Buttock	Extremity	Female	No	Yes	HP Grp 1A	NA	3	Primary	Loss	No
12	27	Leg	Extremity	Female	Yes	Yes	HP Grp 1A	Neurofibroma	2	Primary	Retained	No
13	29	Paraspinal – L4 root	Axial	Male	Yes	Yes	HP Grp 0	Atypical NF	1	Primary	Retained	No
14	23	Leg, upper	Thorax	Female	No	Yes	HP Grp 2	NA	3	Primary	Loss	No
15	43	Thorax	Extremity	Female	No	No	HP Grp 1A	NA	3	Primary	Loss	No
18	18	Sciatic nerve	Axial	Female	Yes	No	HP Grp 1B (W/LG‐areas)	NA	2	Primary	Retained	No
19	93	Chest wall	Extremity	Female	No	No	HP Grp 2	NA	3	Local recurrence	Retained	No
20	60	Thigh	Extremity	Female	No	No	HP Grp 1B (W/pleomorphism)	NA	3	Primary	Loss	No
21	54	Pelvis	Extremity	Female	No	Yes	HP Grp 1B (neurofibroma background)	Neurofibroma	3	Primary	Retained	No
22	61	Elbow	Extremity	Female	Yes	Yes	HP Grp 1A	NA	3	Primary	Loss	No
23	57	Buttock	Extremity	Female	No	Yes	HP Grp 1B (Triton + chondro)	Atypical neurofibroma (different anatomical site)	3	Primary	Retained	No
24	52	Median nerve	Extremity	Female	Yes	Yes	HP Grp 1A	Atypical plexiform NF	3	Primary	Loss	No
26	17	Paraspinal	Axial	Male	Yes	No	HP Grp 2	NA	3	Primary	Retained	No
27	48	Sciatic nerve	Extremity	Female	Yes	Yes	HP Grp 1A	NA	3	Primary	Retained	No
28	29	Thigh	Extremity	Male	No	No	HP Grp 1A	NA	3	Primary	Loss	No
29	59	Calf	Extremity	Female	No	No	HP Grp 2	NA	3	Primary	Retained	No
30	28	Leg, left	Extremity	Female	Yes	Yes	HP Grp 1B (W/LG‐areas)	NA	2	Primary	Retained	No
31	51	Forearm	Extremity	Female	Yes	No	HP Grp 1B	NA	3	Primary	Retained	No
32	41	Thigh	Extremity	Male	No	No	HP Grp 0	NA	1	Primary	Retained	No
33	34	Thigh	Extremity	Male	No	No	HP Grp 2	NA	3	Primary	Retained	No
34	64	Arm	Extremity	Male	No	No	HP Grp 2	NA	3	Primary	Retained	No
35	75	Shoulder	Extremity	Female	No	No	HP Grp 1A (radiation‐associated)	NA	3	Primary	Loss	Yes
36	57	Chest wall	Extremity	Male	No	No	HP Grp 1B (perineural diff.)	NA	3	Primary	Retained	No
37	78	Sciatic nerve	Axial	Female	Yes	No	HP Grp 2	NA	3	Primary	Retained	No
38	62	Thigh	Extremity	Male	No	No	HP Grp 2	NA	3	Primary	Retained	No
39	38	Arm	Extremity	Male	Yes	Yes	HP Grp 1B (Triton)	NA	3	Primary	Loss	No
40	39	Thigh	Extremity	Female	No	Yes	HP Grp 2	NA	3	Primary	Loss	No
41	34	Shoulder	Extremity	Male	No	No	HP Grp 1A	NA	3	Primary	Retained	No
47	36	Thigh	Extremity	Female	No	No	HP Grp 1A	NA	2	Primary	Loss	No
17	69	Leg, lower	Extremity	Female	No	No	HP Grp 6	NA	3	Primary	Retained	No
25	67	Forearm	Extremity	Female	No	No	HP Grp 3	NA	3	Primary	Retained	No
167	11	Forearm	Extremity	Male	No	No	HP Grp 7	NA	3	Primary	Retained	No
168	19	Foot	Extremity	Female	No	No	HP Grp 7	NA	3	Primary	Retained	No
6	54	Shoulder	Extremity	Male	No	No	HP Grp 3	NA	3	Primary	Retained	No
11	73	Thigh	Extremity	Male	No	No	HP Grp 3	NA	3	Primary	Retained	No
160	24	Arm	Extremity	Male	No	No	HP Grp 5	NA	3	Primary	Retained	No
161	46	Buttock	Extremity	Female	No	No	HP Grp 5	NA	3	Primary	Retained	No
16	35	Leg, upper	Extremity	Male	No	No	HP Grp 3	NA	3	Primary	Loss	No
155	73	Thigh	Extremity	Female	No	No	HP Grp 8	NA	NA	Primary	Retained	No
156	61	Axilla	Extremity	Female	No	No	HP Grp 8	NA	NA	Primary	Retained	No
157	84	Chest wall	Extremity	Female	No	No	HP Grp 8	NA	NA	Primary	Retained	No
165	61	Ulnar nerve	Extremity	Female	No	No	HP Grp 4	NA	3	Primary	Retained	No
169	46	Leg	Extremity	Male	No	No	HP Grp 7	NA	3	Primary	Retained	No

* Age (years) at presentation.

### DNA sequencing

Whole‐exome sequencing (WES) or WGS (*n* = 2) was performed on 51 of the 176 tumours for which frozen tumour and matched normal tissue were available (> 60% tumour‐rich and < 20% necrotic). Table 2 provides a summary of the clinicopathological features of these 51 cases. Details for sequencing, data processing, and germline variant classification are provided in supplementary material, Supplementary materials and methods. WES data are available in the EGA database at EMBL‐EBI, https://www.ebi.ac.uk/ega/studies/EGAS0000100452.

### Immunohistochemistry

Immunohistochemistry (IHC) was performed on formalin‐fixed, paraffin‐embedded (FFPE) whole tissue sections cut at 4 μm. Details are provided in supplementary material, Supplementary materials and methods.

### EPIC array protocol and data analysis

DNA methylation array assays were undertaken as published elsewhere [12]. DNA (500 ng) from frozen tumour samples was bisulphite‐converted using a Zymo EZ DNA methylation Gold kit (Zymo Research Corp, Irvine, CA, USA) and hybridised to Infinium HumanMethylationEPIC BeadChip arrays (Illumina, San Diego, CA, USA). Details are provided in supplementary material, Supplementary materials and methods. RNOH and the Heidelberg group methylation array data [12] are available in the ArrayExpress database at EMBL‐EBI under accession number E‐MTAB‐8864.

### Statistical analysis

No statistical methods were used to predetermine sample size. All statistical analyses were performed using R [17]. Kaplan–Meier analysis was conducted using the R packages ‘survminer’ [18] and ‘survival’ [19], with survival time defined as the time from diagnosis to death or last follow‐up: survival data were obtained in August 2019. *P* values less than 0.05 were considered statistically significant.

## Results

### Diagnoses and genomic landscape of MPNST

**Figure 1:**
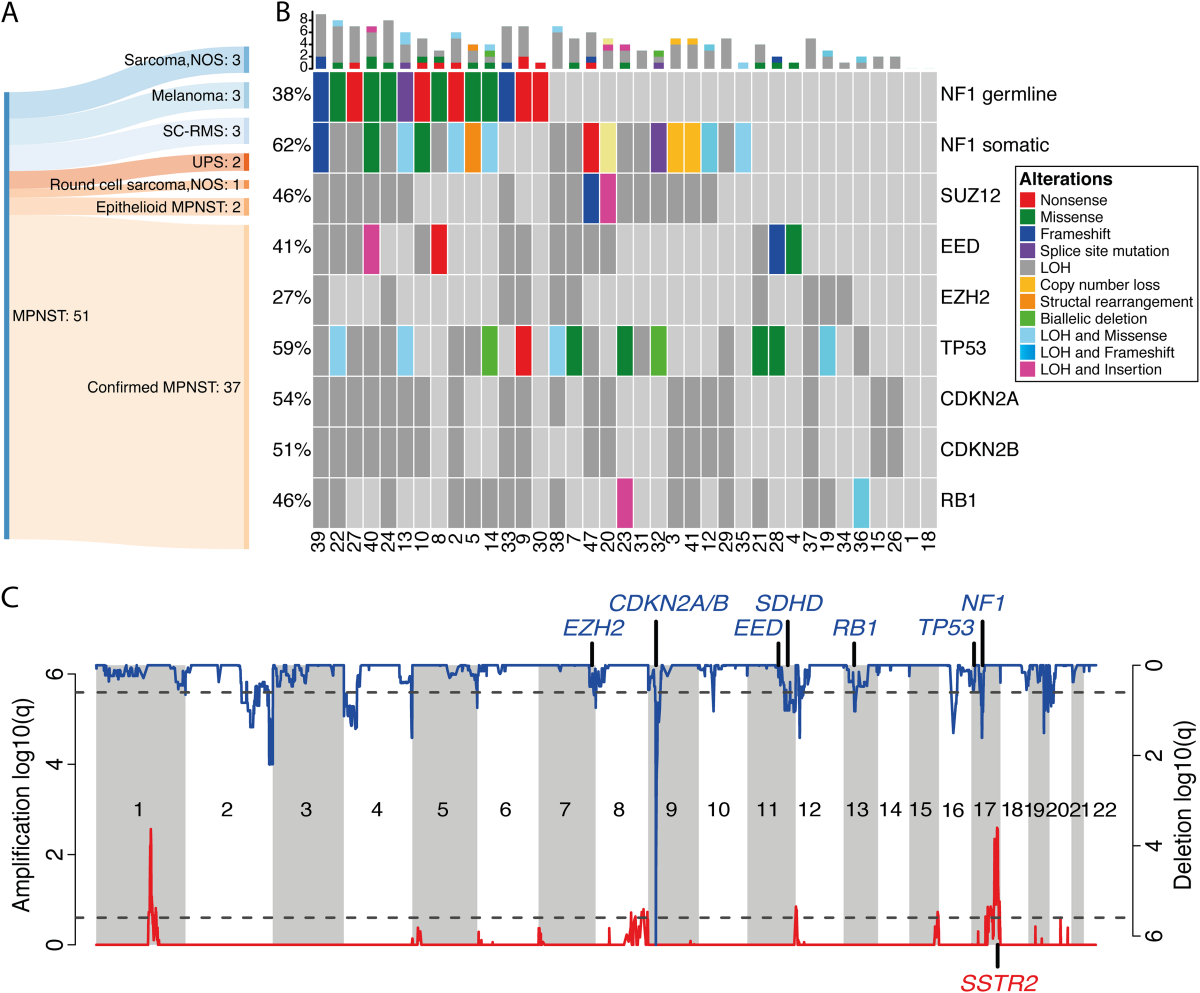
The somatic and germline mutational landscape of 37 MPNSTs. (A) An illustrative summary of the modification to diagnoses resulting from genomic sequencing analysis of 51 MPNSTs. Sarcoma, NOS: spindle cell sarcoma, not otherwise specified; SC‐RMS: spindle cell rhabdomyosarcoma; UPS: undifferentiated pleomorphic sarcoma. (B) OncoPrint showing an overview of the somatic and germline mutations detected in the 37 sequenced MPNST cases. The numbers indicated on the *x*‐axis are the case numbers. Somatic mutations are given for *NF1*, PRC2 components, *TP53*, and *RB1*. The different colours indicate the different types of genomic alterations. The bar plot in the top indicates the numbers of alterations detected for each patient. LOH, loss of heterozygosity, copy number = {0, *n*}, *n* > 0. (C) The CNV landscape of 37 MPNSTs. GISTIC plots showing regions of recurrent copy number gains (red) or copy number losses (blue) of the sequenced MPNST cases.

The diagnosis of MPNST was excluded in 12/51 (27%) of the sequenced tumours following review of the pathology, new IHC, molecular tests, and clinical information. One sample (case 16, reclassified as UPS) failed the sequencing quality control (Figure 1A, Table 2, and supplementary material, Table S1 show details of the refined diagnoses).

### *NF1* mutations in MPNST

Of 39 of the 51 sequenced tumours considered to represent MPNST, two were epithelioid MPNSTs and 16 were from patients exhibiting the clinical phenotype of NF‐1, a finding substantiated by detection of germline *NF1* mutations in 12/16 patients (Table 2, supplementary material, Table S1, and Figure 1B). Nevertheless, the tumours in the four remaining patients (cases 4, 12, 21, and 23) were considered to represent *bona fide* MPNSTs on the following evidence: all had an NF‐1 clinical phenotype; the tumour from case 12 arose from a large nerve and exhibited a low‐grade component; and cases 21 and 23 contained a low‐grade component, while case 4 revealed heterozygous alterations of core genes of PRC2. Of the remaining 21 tumours, three harboured biallelic somatic *NF1* alterations; two had homozygous *NF1* copy number loss (detected by visual inspection of read counts using the Integrative Genomics Viewer); three had non‐synonymous *NF1* mutations; and two had a heterozygous loss of *NF1*. In total, 27/37 (73%) MPNSTs had *NF1* genetic alterations including loss of heterozygosity (LOH) (Figure 1B and Table 2). Of the remaining ten MPNSTs in which an *NF1* alteration was not detected, six exhibited typical morphological features (cases 1, 15, 18, 21, 28, and 36) and were categorised in HP Group 1A or 1B (supplementary material, Figure S1). Four tumours had a higher degree of nuclear pleomorphism than seen in the MPNST classical variant (HP Group 2), three of which (cases 19, 34, and 37) harboured LOH in *EZH2*. Neither *NF1* nor PRC2 alterations were detected in case 26 (H3K27me3‐retained), but this case arose from a paraspinal nerve, as did case 37.

### Whole‐genome doubling and copy number alterations are common in MPNST

We observed evidence of genome doubling (GD) in 25/37 (68%) tumours, including 2 × GD in two cases (cases 7 and 30) (supplementary material, Table S3 and Figure S2). 22/25 (88%) tumours with GD were grade 3, but GD was not associated with a particular histological phenotype or anatomical site (Table 2). Recurrent regions of gain and loss included amplification in six genomic regions, most reported previously [20, 21, 22]. Notably, the region 17q25.1 (chr17:70118936–72241393), which contains the somatostatin receptor 2 (*SSTR2*), was amplified in 12/37 (32%) cases (supplementary material, Table S5 provides a list of genes in this region). Sixteen genomic regions showed recurrent loss including the genomic loci containing *NF1*, *SUZ12* (17q11.2), and *CDKN2A/B* (9p21.3) (Figure 1C and supplementary material, Table S5).

### At least 50% of MPNSTs have alterations in PRC2‐related genes

DNA sequencing of MPNSTs revealed non‐synonymous somatic mutations in *EED* (4/37; 11%) and *SUZ12* (2/37; 5%). None of the 37 tumours displayed somatic mutations in *EZH2*. LOH was observed in *EED* in 12/37 (32%), in *SUZ12* in 16/37 (43%), and in *EZH2* in 10/37 (27%) cases (Figure 1B), and *TP53* was lost in 17/37 (46%) cases. Two tumours, cases 20 and 40, had a somatic mutation in addition to LOH (biallelic alterations) in *EED* and *SUZ12*, respectively. In total, 25/37 (68%) cases had at least one genomic aberration in *EED*, *SUZ12* or *EZH2* (including LOH) (Table 3 and Figure 1B). Alterations in PRC2 core components were detected in 13/16 (81%) tumours in the setting of NF‐1 and in 12/21 (57%) sporadic tumours. For completeness, visual inspection of other PRC2 complex genes, *EPC1*, *AEBP2*, and *RBBP7*, was undertaken but revealed no non‐synonymous mutations. One case had a missense mutation in the H3K27me3 demethylase KDM6B (case 32, supplementary material, Table S2). A driver mutation analysis (supplementary material, Supplementary materials and methods) confirmed the importance of the PRC2 genes in MPNST by classifying *EED* and *NF1* as significant driver genes.

**Table 3:** Genomic and immunohistochemistry profiles of 37 sequenced MPNST cases.

Case	NF1 phenotype	*NF1* germline mutation	*NF1* somatic mutations	*SUZ12* somatic mutations	*EED* somatic mutations	*EZH2* somatic mutations	*TP53* somatic mutations	H3K27me3 IHC status	Genome doubling	Multiple genome doubling
1	No	Normal	Normal	Normal	Normal	Normal	Normal	Retained	False	False
2	Yes	c.5902C>T	Missense c.1658A>G	Normal	Normal	Normal	LOH	Retained	False	False
3	No	Normal	Copy number loss	LOH	Normal	Normal	Normal	Loss	True	False
4	Yes	Normal	LOH	Normal	Missense c.715G>T	Normal	Normal	Loss	True	False
5	No	Normal	Structural rearrangement + LOH	Normal	Normal	Normal	LOH	Retained	False	False
7	No	Normal	LOH	LOH	LOH	LOH	LOH and missense c.1009C>T	Retained	True	True
8	Yes	c.3233C>G and c.3779T>C	Normal	Normal	Nonsense c.58A>T	Normal	Normal	Loss	True	False
9	Yes	c.574C>T	Normal	Normal	LOH	LOH	Nonsense c.592G>T	Retained	True	False
10	Yes	c.3200A>T	Missense	Normal	LOH	Normal	Normal	Loss	True	False
12	Yes	Normal	Nonsense,c.121G>T + LOH	LOH	Normal	Normal	Normal	Retained	True	False
13	Yes	Splice site mutation in exon 40 (G>T)	Missense, c.6425G>A + LOH	LOH	Normal	Normal	LOH and missense c.404G>A	Retained	False	False
14	Yes	c.3200A>T	Missense, c.6343C>T + LOH	Normal	Normal	Normal	Biallelic deletion	Loss	False	False
15	No	Normal	Normal	Normal	Normal	Normal	Normal	Loss	True	False
18	No	Normal	Normal	Normal	Normal	Normal	Normal	Retained	False	False
19	No	Normal	Normal	Normal	Normal	LOH	LOH and nonsense c.281C>G	Retained	True	False
20	No	Normal	c.5285_5345del61 + LOH	LOH and p.R196fs*4 Del (fs)	LOH	Normal	Normal	Loss	True	False
21	Yes	Normal	Normal	Normal	LOH	LOH	Missense c.833C>A	Retained	True	False
22	Yes	c.2533T>C	LOH	LOH	LOH	Normal	LOH and missense c.843C>A	Loss	True	False
23	Yes	Normal	LOH	LOH	LOH	Normal	Missense c.843C>A	Retained	True	False
24	Yes	c.5102A>G	LOH	LOH	Normal	LOH	LOH	Loss	True	False
26	No	Normal	Normal	Normal	Normal	Normal	Normal	Retained	False	False
27	Yes	c.147C>A	LOH	LOH	Normal	Normal	LOH	Retained	True	False
28	No	Normal	Normal	Normal	Frameshift,c.1157delT	Normal	c.404G>A missense	Loss	True	False
29	No	Normal	LOH	Normal	Normal	Normal	LOH	Retained	True	False
30	Yes	c.7348C>T	Germline mutation.	Normal	Nonsense c.58A>T	Normal	LOH	Retained	True	True
31	No	Normal	LOH	LOH	Normal	Normal	LOH	Retained	False	False
32	No	Normal	c.803_888+10del96, splice site + LOH	LOH	Normal	Normal	Biallelic deletion	Retained	False	False
33	No	Normal	LOH	LOH	LOH	LOH	Normal	Retained	True	False
34	No	Normal	Normal	Normal	Normal	LOH	Normal	Retained	True	False
35	No	Normal	Missense c.1885G>A	Normal	Normal	Normal	Normal	Loss	True	False
36	No	Normal	Normal	Normal	Normal	Normal	LOH	Retained	True	False
37	No	Normal	Normal	Normal	Normal	LOH	LOH	Retained	True	False
38	No	Normal	LOH	LOH	LOH	LOH	LOH + missense c.711G>A	Retained	True	False
39	Yes	c.7099_7102delCTGG	Frameshift, c.4478_4479ins	LOH	Normal	LOH	LOH	Loss	False	False
40	Yes	c.1112A>G	c.1804G>A, missense + LOH	LOH	Frameshift,c.746delA, LOH	Normal	LOH	Loss	False	False
41	No	Normal	Structural rearrangement	LOH	Normal	Normal	Normal	Retained	False	False
47	No	Normal	Nonsense, c.7906C>T	Frameshift p.K395fs*7	LOH	Normal	LOH	Loss	True	False

We investigated how H3K27me3 immunoreactivity correlated with the genomic data. Loss of the protein was observed in all cases (*n* = 6) in which an SNV in any of the PRC2 genes was detected (*SUZ12*, *EED*; *p* = 0.004, chi‐square independence test). However, eight cases showed loss of H3K27me3 without an SNV being detected at these genes, although five of these had LOH in either *SUZ12* or *EED*. In contrast, immunoreactivity was not associated with LOH of *EZH2*. In summary, loss of H3K27me3 expression was observed in 50% (*n* = 16) of NF‐1‐associated MPNSTs, 25% (*n* = 20) of sporadic MPNSTs, and in the single radiation‐associated MPNST (case 35, Table 3), with an overall loss of H3K27me3 in 38% (14/37) of sequenced MPNSTs, including two grade 1 cases.

### MPNST histology correlates with H3K27me3 immunoreactivity

**Figure 2:**
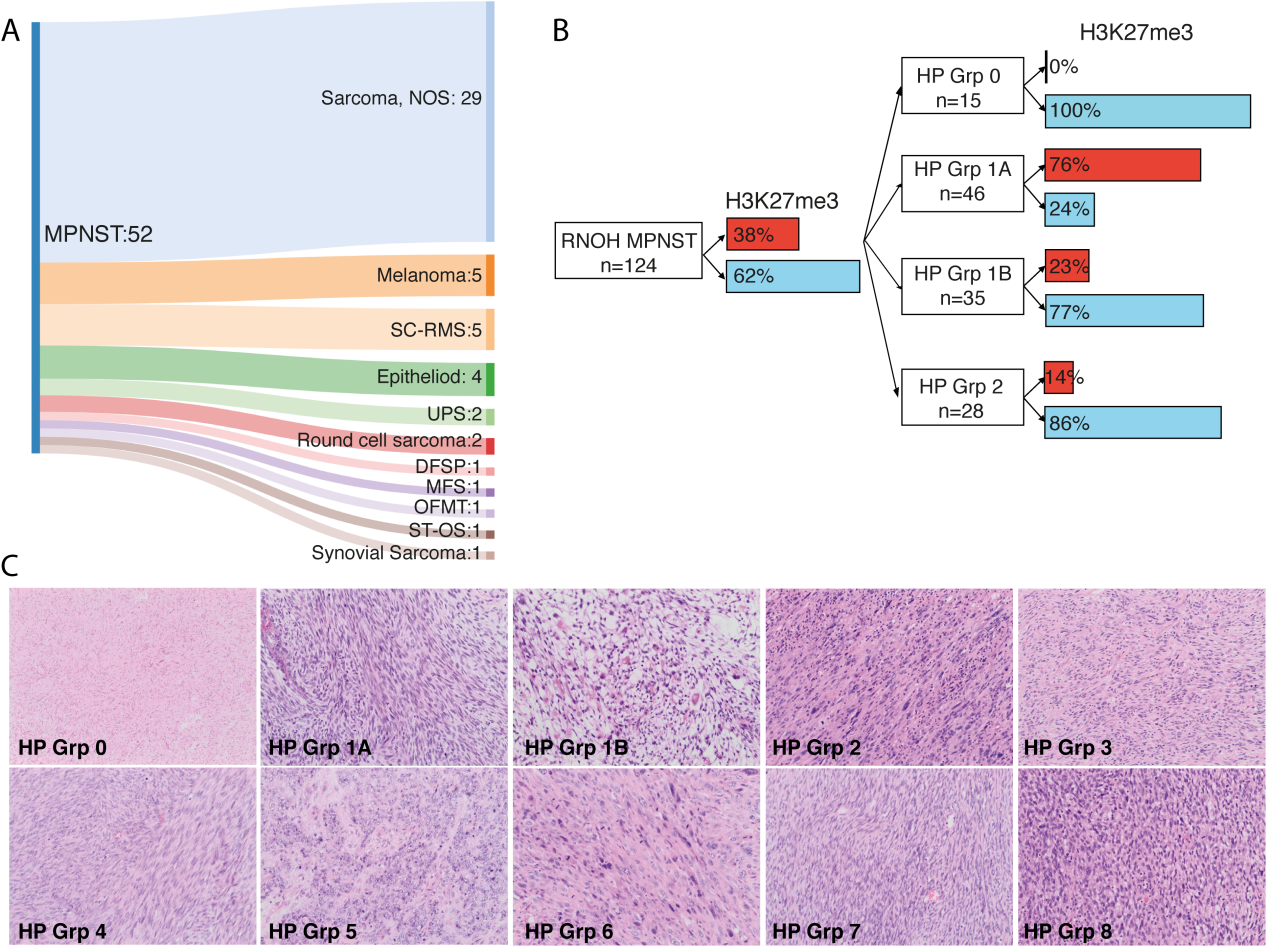
Histopathological groups and their correlation with H3K27me3 immunoreactivity status. (A) A Sankey plot showing the modification to diagnoses made in 52/176 of the tumours where a diagnosis of MPNST was provided or was considered in the differential diagnosis. Sarcoma, NOS: spindle cell sarcoma, not otherwise specified; SC‐RMS: spindle cell rhabdomyosarcoma; UPS: undifferentiated pleomorphic sarcoma; DFSP: dermatofibrosarcoma protuberans; OFMT: ossifying fibromyxoid tumour; ST‐OS: soft‐tissue osteosarcoma. (B) The H3K27me3 immunoreactivity status of 124 RNOH MPNST cases. 38% of the entire cohort exhibited loss of H3K27me3 expression. Correlation of the H3K27me3 status with HP Grps 0, 1A, 1B, and 2 is indicated. Red = loss of H3K27me3; blue = retained H3K27me3 expression. (C) The ten histological subgroups. **HP Grp 0**: case 13. Low‐grade MPNST (H&E, ×10). **HP Grp 1A**: case 15. Classical MPNST, grade 3, showing a tumour with a compact fasciculated architecture composed of monomorphic spindle cells with tapering nuclei (H&E, ×10). **HP Grp 1B**: case 22. MPNST with heterologous rhabdomyoblastic differentiation (Triton tumour). Note the plump cells with brightly eosinophilic cytoplasm and striations, indicative of a rhabdomyoblastic phenotype. These cells were strongly immunoreactive for desmin and myogenin (H&E, ×10). **HP Grp 2**: case 51. A spindle cell sarcoma arising from a nerve in a patient with neurofibromatosis type 1. The morphological features are not entirely classical but the associated clinicopathological features support the diagnosis (H&E, ×10). **HP Grp 3**: case 6. Spindle cell sarcoma, NOS. The morphological features are not typical for MPNST and there was no other supporting evidence to justify the diagnosis of MPNST (H&E, ×10). **HP Grp 4**: case 83. A tumour with definitive molecular evidence to exclude the diagnosis of MPNST. This case is a dermatofibrosarcoma protuberans with fibrosarcomatous transformation, confirmed by the presence of a *COL1A1–PDGFB* fusion on FISH (H&E, ×10). **HP Grp 5**: case 116. A tumour composed of sheets of cells with an epithelioid morphology. This tumour was strongly positive for S100 and has loss of INI‐1 (H&E, ×10). **HP Grp 6**: a tumour composed of pleomorphic spindle cells and an undifferentiated immunophenotype, confirming the diagnosis of undifferentiated pleomorphic sarcoma (H&E, ×10). **HP Grp 7**: case 7. A tumour composed of monomorphic spindle cells with eosinophilic cytoplasm, embedded in a densely collagenous stroma. This tumour was strongly positive for desmin and myogenin, and had a *MYOD1* p.L122R missense mutation, confirming the diagnosis of spindle cell/sclerosing rhabdomyosarcoma (H&E, ×10). **HP Grp 8**: case 17. A pleomorphic spindle cell tumour, focally positive for S100 and negative for melan‐A and HMB45 with a mutational signature attributable to UV light, supporting the diagnosis of malignant melanoma (H&E, ×10).

To investigate the variable loss of H3K27me3 immunoreactivity reported in previous studies [3, 4, 8, 9, 11, 13], we extended our tumour set to 176 cases, including the above‐mentioned 51 sequenced cases. Following a review of the pathology, combined with additional molecular diagnostic testing and clinical information, we refined our original diagnoses in 30% (*n* = 52/176) of cases (Figure 2A and supplementary material, Table S1). Of the remaining 124 MPNSTs, 38% (*n* = 47/124) of MPNSTs revealed loss of H3K27me3 immunoreactivity. However, when classified according to the HP groups, we found that loss of H3K27me3 expression was observed in 76% (35/46 cases) of HP Group 1A, and 0/15, 23% (8/35), and 14% (4/28) of cases in HP Groups 0, 1B, and 2, respectively (Figure 2B). Loss of H3K27me3 expression was seen in all five radiotherapy‐associated MPNSTs, whereas it was retained in 76% (16/21) of paraspinal cases (supplementary material, Table S1 and Figure S3), in the two epithelioid MPNSTs, and in all but 2/52 non‐MPNST high‐grade sarcomas cases (case 16, UPS; case 5, sarcoma NOS – HP Group 3). Loss of H3K27me3 expression was seen in 40% (19/47) of patients with an NF‐1 phenotype and in 32% (22/69) of sporadic cases (supplementary material, Tables S1 and S3). Assessment of 82 synovial sarcomas, harbouring the characteristic fusion gene, and 53 UPSs revealed loss of H3K27me3 immunoreactivity in only two cases and one case, respectively [15] (supplementary material, Table S4). Three per cent (5/187) of low‐grade MPNST cases revealed loss of H3K27me3 calculated as a specificity of 97% (182/187) and a sensitivity of 43% (47/109).

In view of the demethylation role of KDM6A/B, these loci were interrogated but revealed no evidence of biallelic inactivation; no association was found between the loss of copy number of these genes and the expression of H3K27me3 (supplementary material, Table S1).

### Nerve sheath tumours are characterised by distinct methylation patterns

Loss of H3K27me3 expression caused by genetic alterations of the PRC2 genes is associated with a global change in methylation levels, a finding reflected in the Heidelberg publication [12]. This prompted us to analyse our DNA methylation profiles from the 102 cases from which DNA was available. We included the DNA methylation profiles of 53 previously reported UPS cases [15], thereby allowing comparison with 29 high‐grade sarcomas NOS included in our 176 cases (supplementary material, Figure S4) and the Heidelberg methylation dataset: in total, a dataset from 309 cases.

**Figure 3:**
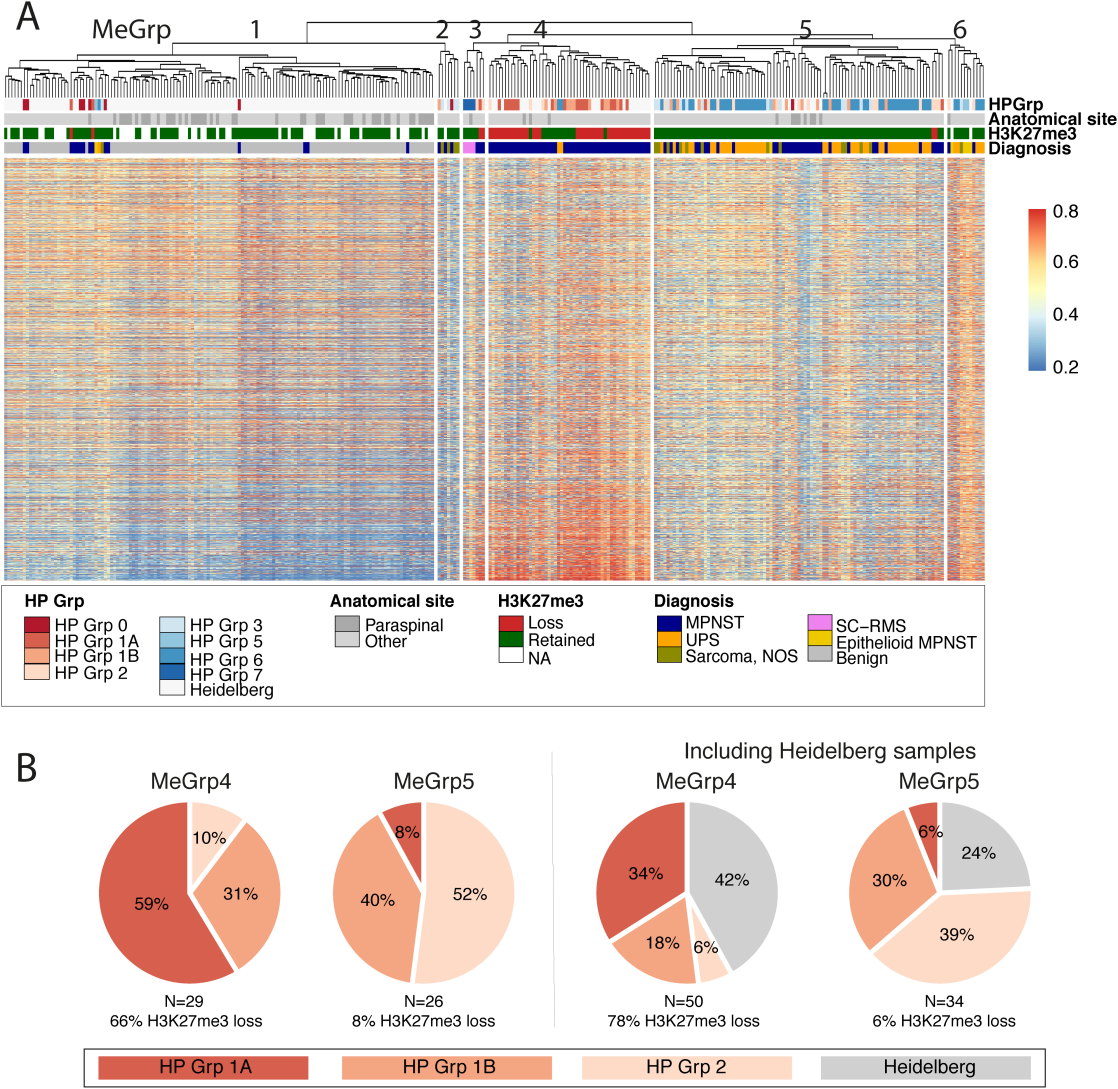
Methylation profiling identifies distinct MPNST groups. (A) Unsupervised hierarchical clustering showing 309 samples from RNOH and Heidelberg clustered in epigenetically distinct subgroups of benign nerve sheath tumours, low‐grade MPNSTs, high‐grade MPNSTs, UPSs, epithelioid MPNSTs, and spindle cell rhabdomyosarcoma (SC‐RMS) subtypes. MPNSTs are clustered in methylation groups (MeGrps) 4 and 5. MeGrp 4 contains MPNSTs showing loss of H3K27me3 and hypermethylation of PRC2 pathway genes as opposed to MeGrp 5, which contains MPNST and UPS samples with retained H2K37me3 expression. The majority of the paraspinal tumours clustered in MeGrp 5. MeGrp 1 is composed largely of benign nerve sheath tumours and low‐grade MPNSTs. MeGrp 2 consists mostly of sarcoma NOS samples (not sequenced), whereas MeGrp 3 contains mostly SC‐RMS cases. MeGrp 6 contains the majority of epithelioid MPNSTs. (B) Pie chart showing the association between the MPNSTs in MeGrps 4 and 5 and the histopathological groups (HP Grps) 1A, 1B, and 2. The histopathology of the Heidelberg cases has not been reviewed in this study.

Unsupervised hierarchical clustering of the combined RNOH and Heidelberg cases revealed six methylation groups (MeGroups 1–6, Figure 3A and supplementary material, Table S7): 85% of all high‐grade MPNSTs clustered in MeGroups 4 and 5; 78% of MeGroup 4 cases (39/50) revealed loss of H3K27me3 protein, whereas 94% (31/33) of cases in MeGroup 5 retained H3K27me3 expression. The latter also contained 57% (46/53) of UPSs and 66% (12/18) of sarcomas NOS. Notably, 8/14 paraspinal MPNSTs formed a distinct cluster within MeGroup 5. Overall, MeGroup 4 comprised largely HP Group 1A MPNSTs, all with loss of H3K27me3 expression, whereas MeGroup 5 was dominated by HP Group 1B and HP Group 2 samples (Figure 3B).

MeGroup 1 (*n* = 138) contained all benign and low‐grade nerve sheath samples from Heidelberg and RNOH, but also included six high‐grade sarcomas. MeGroup 2 comprised sarcoma NOS samples (*n* = 4) and three MPNSTs (cases 32, 289, and one Heidelberg sample), all of which retained H3K27me3 expression. MeGroups 3 and 6 contained all three SC‐RMS and the majority of the epithelioid MPNSTs, respectively (Figure 3A and supplementary material, Table S7).

Finally, the methylation data available from two of our five reclassified malignant melanoma cases were studied in combination with the TCGA melanoma methylation data. A t‐SNE analysis supported a diagnosis of melanoma (supplementary material, Figure S6).

### Polycomb target genes are differentially methylated between the two MPNST methylation groups

We analysed the differentially methylated regions (DMRs) and probes (DMPs) between MPNSTs in MeGroup 4 (*n* = 50) and MeGroup 5 (*n* = 33), of which 12 and 15 had been exome‐sequenced, respectively. Methylation analysis revealed a total of 606 DMRs and 123,950 DMPs between MPNSTs in these two groups, of which 85,601 showed increased methylation in MeGroup 4 (supplementary material, Tables S8 and S9). Gene Set Enrichment Analysis (GSEA) showed strong enrichment for pathways associated with PRC2 biology (supplementary material, Table S10).

Analysis revealed that 1,200 of 1,350 (89%) possible PRC2 target genes (supplementary material, Supplementary materials and methods) were differentially methylated between the two MeGroups, 65% of which (784/1,200 target genes) were hypermethylated in the promoter regions (200 or 1,500 bp upstream of the transcription start site, TSS200 and TSS1500, respectively), indicating that the PRC2 target genes are not expressed in MeGroup 4. Analysis of the exome profiles failed to identify alterations that distinguished cases from the two MeGroups.

Finally, when comparing only cases with retained H3K27me3 from MeGroup 4 (*n* = 11) and MeGroup 5 (*n* = 32), we found that 269 DMRs and 10,862 DMPs significantly separated these cases (supplementary material, Tables S11 and S12). 9042/10,862 DMPs were more methylated in the H3K27me3‐positive cases in MeGroup 4 than in MeGroup 5, and 9,615 of these 10 862 DMPs were also differentially methylated in samples in the two groups. Of the 1,350 PRC2 target genes, the majority (89%) were differentially methylated between all samples in the two MeGroups, whereas only 409 were differentially methylated, when analysing only those samples which retained H3K27me3, of which 191 had hypermethylation of promoter regions. GSEA showed strong enrichment for terms associated with PRC2 biology (supplementary material, Table S13).

### H3K27me3 and genome doubling (GD) status as prognostic markers of survival

No correlation was found between survival and patients in MeGroups 4 (*n* = 28) and 5 (*n* = 25) (*p* = 0.71, supplementary material, Figure S7A). There was also no association between H3K27me3 immunoreactivity status and survival in MeGroups 4 and 5 (*p* = 0.88, supplementary material, Figure S7B), a finding validated on extending the analysis to 100 RNOH patients with high‐grade MPNST for which survival data were available (*p* = 0.94, supplementary material, Figure S8 and Table S14).

Given a recently published paper highlighting that GD is associated with poor prognosis across cancer types [23], we asked if such genomic events in MPNST correlated with clinical outcome. This analysis was performed on the 37 sequenced MPNSTs: GD was observed in neither of the two cases in HP Group 0, in 91% (10/11) of HP Group 1A, in 55% (6/11) of HP Group 1B, and in 69% (9/13) of HP Group 2. Despite a strong tendency, GD status and survival did not reach statistical significance (*p* = 0.06, supplementary material, Figure S9 and Table S14).

### A subset of MPNSTs express SSTR2

In view of the lack of effective treatments for MPNST, we sought potential therapeutic targets. Our genomic data revealed that *SSTR2* was amplified in 32% of cases and FISH confirmed amplification in 8/11 of these cases (supplementary material, Table S15 and Figure S10). The discrepancy in the three cases may be accounted for by tumour heterogeneity; moreover, these cases were also aneuploid, making interpretation of the FISH challenging. Two of ten cases for which RNA‐sequencing data were available showed increased expression of *SSTR2* transcripts compared with the other eight cases (supplementary material, Figure S11). However, protein expression (SSTR2 IHC, supplementary material, Figures S12 and S13) showed little correlation with amplification on FISH or sequencing (supplementary material, Table S15), with only 12/55 (22%) cases being immunoreactive for SSTR2, only four of which were *SSTR2*‐amplified on WES and five by FISH. The expression of SSTR2 protein was neither associated with any of the HP Groups nor associated with the MeGroups (supplementary material, Table S15; SSTR2 IHC, Figure S14).

## Discussion

In this study of well‐characterised high‐grade MPNSTs, we find that loss of H3K27me3 expression, occurring in approximately 40% of cases, is lower than reported by most groups, but similar to that reported by Bovée and co‐workers [8]. We identified that H3K27me3 expression was largely dictated by the morphology of the tumours: 76% of the MPNSTs exhibiting the classical histological features (HP Group 1A) revealed loss of H3K27me3 expression, whereas MPNSTs with heterologous elements or with a low‐grade component (HP Group 1B) revealed loss of H3K23me3 in just 23% of cases. Lastly, in those MPNSTs where a diagnosis could not be proffered confidently on morphology alone (HP Group 2), H3K27me3 loss was detected in only 14% of cases. We conclude that the variation of reported loss of H3K27me3 immunoreactivity is dictated by the different histological subgroups within MPNSTs, and propose that the reported differences in the frequency of H3K27me3 loss by others could be explained by the number of these subtypes represented in the different studies [3, 4, 9, 10, 11]. Although our findings show that loss of expression of this marker is highly specific for MPNST, we consider that its sensitivity is variable and dependent on subtypes described herein.

Consistent with previous publications, all of our radiation‐associated MPNST cases showed loss of H3K27me3 expression [3]. However, in contrast to other reports [3, 12], we did not find any association between H3K27me3 loss with germline or sporadic cases, and six of our paraspinal cases revealed H3K27me3 loss, three of which were associated with radiation therapy. Differences in H3K27me3 immunohistochemistry results across laboratories could reflect how the IHC was interpreted: ‘partial loss’ of the protein has been reported [13], whereas we reported cases as ‘positive’ or ‘negative’. However, the number of cases reported with partial loss in the literature is unlikely to account fully for the wide variability in the published results.

Röhrich *et al* were the first to report the association between the methylation status of H3K27me3 and the immunoreactivity of this chromatin mark in MPNST. Our data confirm previous publications linking genetic alterations in one of the PRC2 complex components to tri‐methylation of lysine 27 and loss of the protein. Methylation analysis of our combined data revealed, as reported previously [12], a distinct cluster containing atypical neurofibroma and low‐grade MPNSTs (MeGroup 1), although six high‐grade sarcomas were also in this cluster, highlighting that optimisation of the classifier is required prior to introducing methylation profiling into a diagnostic clinical service. The majority of high‐grade MPNSTs formed two methylation groups, MeGroups 4 and 5, with Heidelberg and RNOH cases distributed across both MeGroups. MeGroup 4, composed largely of MPNSTs with classical features (HP Group 1A), was characterised by hypermethylation of PRC2 target genes and loss of H3K27me3 immunoreactivity in 78% of cases. In contrast, MeGroup 5 was composed of HP Group 2 cases, of which 94% retained H3K27me3 expression. Notably, most paraspinal MPNSTs clustered together within MeGroup 5, but did not form a distinct cluster as previously reported [12].

It is noteworthy that 30% of cases in MeGroup 4, which is composed largely of the classical variant of MPNSTs, show retention of H3K27me3 positivity. It is remarkable that these cases also showed a strong enrichment for GSEA terms associated with PRC2 biology similar to those cases in which H3K27me3 expression was lost. We speculate that this finding could be explained by activation of the non‐canonical PRC2 pathway, a biological process that is well described in the regulation of gene transcription [24]. For example, there is considerable evidence that EZH2, the enzymatic subunit of PRC2, can act independently of the PRC2 complex, and in an independent enzymatic manner, to bring about transcriptional regulation through a variety of epigenetic mechanisms. This has in some cases been shown to involve non‐core PRC2 proteins such as AE binding protein 2, JARID2, PHD finger protein 19, and long non‐coding RNAs including HOTAIR and microRNAs, some of which have already been implicated in dysregulation of gene transcription in disease [25, 26].

Virtually all MPNSTs in MeGroup 5, in contrast to MeGroup 4, expressed H3K27me3 and the morphology of the two groups is striking. MeGroup 5 tumours are often less clearly recognised as nerve sheath in origin. Furthermore, the clustering of these MPNSTs with UPS in MeGroup 5 underscores the morphological spectrum of tumours in this MeGroup and the difference to those in MeGroup 4. Perturbation of physiological and developmental processes that are normally tightly co‐ordinated through the regulation of expression and repression of genes forming PRC2, a multi‐subunit epigenetic protein complex, could potentially account for these findings [27]. It is therefore interesting to speculate that the differences in MPNSTs in MeGroups 4 and 5 reflect their cell of origin and path of differentiation. However, the factors that determine the development of nerve sheath tumours rather than UPS remain unknown. Furthermore, the molecular events that promote transformation of MPNST to high‐grade disease in MeGroup 5 remain to be determined.

The detection of *SSTR2* amplification in 30% of our sequenced MPNSTs, which is supported by FISH and RNA sequencing but not immunoreactivity, is novel and of clinical interest, because somatostatin receptor antagonists are available in clinical practice [28]. However, the biological impact of SSTR2 is unclear; with evidence in the literature supporting both a pro‐tumour survival signal and cancer growth inhibition [29]. In small cell lung cancer, there is an association between SSTR2 expression and poor outcome [29], whereas in this small cohort of patients with MPNSTs, we found no link between outcome and SSTR2 amplification status.

We have shown that reaching a diagnosis of MPNST is challenging, but accuracy is improved by excluding mimics with recognised molecular hallmarks, such as *SYT–SSX* fusion genes in synovial sarcoma. We consider that the most rigorous means of classifying MPNST is identification of biallelic *NF1* alterations, which although not specific for MPNST, are considered diagnostic in the context of the relevant clinical/pathology context. However, the identification of all *NF1* functional alterations is challenging because of the large gene size (60 exons), the absence of mutational hotspots, the presence of large structural alterations, and *NF1* germline cryptic splice site variants [30], and is best achieved using WGS and RNA sequencing. Hence, despite our best efforts to classify MPNSTs correctly using WES, we accept that a rare case may not be an MPNST, and that occasional MPNSTs may have been classified as sarcoma NOS. The use of WES in this study is also likely to explain why *NF1* germline alterations were identified in only 71% of patients with the NF‐1 phenotype. Future large‐scale comprehensive multi‐omics analyses, and functional studies of MPNSTs and those suspected of being MPNST should provide a strong evidence base for classifying this disease and understanding its pathogenesis [31].

## Author contributions statement

AMF was responsible for study design. DL, AMF, AMR, HY and CD collected data. IL, DL, CDS, PL, ACS, NP and AMF analysed and interpreted data. AMF, RT, DL and FA were responsible for pathology review. IL, AMF and DL wrote the manuscript, with contributions from NP and RT. All the authors reviewed and edited the paper.

## Supporting information


**Supplementary materials** **and methods**

**Figure S1.** Histopathological features of the ten MPNSTs showing no germline or somatic mutation in *NF1*
**Figure S2.** Whole‐genome doubling estimation
**Figure S3.** Paraspinal case with loss of H3K27me3 immunoreactivity
**Figure S4.** Hierarchical clustering of RNOH cases
**Figure S5.** MeGrp 2: histopathological features
**Figure S6.** Malignant melanoma cases
**Figure S7.** No correlation between MeGroups or H3K27me3 status and survival
**Figure S8.** H3K27me3 loss is not associated with inferior survival in MPNST
**Figure S9.** Cases with genome doubling have a tendency towards shorter overall survival
**Figure S10.** Representative images of *SSTR2* FISH analysis
**Figure S11.** RNA sequencing confirms higher expression of *SSTR2* in samples with genomic amplification of *SSTR2*
**Figure S12.** SSTR2 immunohistochemistry (IHC) controls
**Figure S13.** Representative images of SSTR2 IHC

**Table S1.** Clinicopathological features and reclassification of cases (*n* = 176)
**Table S2.** SNVs from 37 sequenced MPNST cases
**Table S3.** Estimation of whole‐genome doubling (GD)
**Table S4.** H3K27me3 status of 82 synovial sarcoma cases
**Table S5.** GISTIC deleted/amplified regions, 37 sequenced MPNST cases
**Table S6.** SNVs from two sequenced epithelioid MPNST cases
**Table S7.** Methylation groups of 387 cases included in this study
**Table S8.** Differential methylated regions (DMRs) between MPNSTs in methylation groups 4 and 5
**Table S9.** Differential methylated probes (DMPs) between MPNSTs in methylation groups 4 and 5
**Table S10.** GSEA analysis – methylation group 4 versus 5
**Table S11.** Differential methylated regions (DMRs) between H3K27me3 retained cases in methylation groups 4 and 5
**Table S12.** Differential methylated probes (DMPs) between retained H3K27me3 MPNSTs in methylation groups 4 (*n* = 11) and 5 (*n* = 34)
**Table S13.** GSEA analysis – retained H3K27me3 MPNST cases in methylation groups 4 and 5
**Table S14.** Survival in relation to H3K27me3 IHC status and genome doubling (GD) of RNOH MPNST cases
**Table S15.** SSTR2 analysis

## Data Availability

Methylation array data are available in the ArrayExpress database at EMBL‐EBI under accession number E‐MTAB‐8864 (https://www.ebi.ac.uk/arrayexpress/experiments/E‐MTAB‐8864/). WES data and RNA‐sequencing data are available in the EGA database at EMBLEBI, https://www.ebi.ac.uk/ega/datasets/EGAD00001006253.
