# Supplementary material for: H3K27me3 expression and methylation status in histological variants of malignant peripheral nerve sheath tumours
Source: J Pathol. 2020 Sep 1;252(2):151–64. doi: 10.1002/path.5507 (PMC8432159; doi:10.1002/path.5507)
Supplement: Supplementary file 1 — Supplementary materials and methods [file PATH-252-151-s003.docx]

**H3K27me3 expression and methylation status in histological variants of malignant peripheral nerve sheath tumours**

I Lyskjær *et al. J Pathol* DOI: 10.1002/path.5507

**Supplementary materials and methods**

Reference numbers refer to the main text list

*Reclassification of cases*

A total of 176 cases with a diagnosis of ‘MPNST’ in accordance with the WHO criteria from 1 January 2003 to 21 December 2015 were reviewed, and where a differential diagnosis was considered possible, further tests were requested to confirm the suspected diagnosis. Cases with a relatively monomorphic spindle cell morphology and strong expression of desmin and myogenin were screened and those found to have a somatic pL122R *MYOD1* mutation were reclassified as spindle cell/sclerosing rhabdomyosarcoma. Furthermore, cases with the characteristic morphological features of spindle cell/sclerosing rhabdomyosarcoma but without a pL122R *MYOD1* somatic mutation were also reclassified as such. Epithelioid MPNSTs were determined based on epithelioid morphology and loss of *INI-1* expression on IHC. One case harboured an *SS18* gene rearrangement, detected by FISH, and was reclassified as synovial sarcoma (case 173). One case had a *COL1A1–PDGFB* and was reclassified as a DFSP (case 172). Two of the tumours had a ‘hypermutational phenotype’ detected on WES with associated UV-mutational signatures and both presented in an axillary location, resulting in reclassification as melanoma (cases 156 and 157). A further three tumours (cases 155, 158, and 159) had methylation characteristics similar to melanoma, and on morphological review, were also reclassified as such. The following tumours were reclassified on the basis of review of the morphological appearances: 29 as sarcoma, not otherwise specified; two round cell sarcomas (not otherwise specified, cases 165 and 171); two undifferentiated pleomorphic sarcomas (UPS, cases 16 and 17); one myxofibrosarcoma (case 174); one ossifying fibromyxoid tumour (OFMT, case 170), and one soft tissue osteosarcoma (ST-OS, case 175) (supplementary material, Table S1).

*Immunohistochemistry*

Immunohistochemistry (IHC) was performed on 4-μm-thick formalin-fixed, paraffin-embedded (FFPE) whole tissue sections. Diaminobenzidine (DAB) was utilised for visualisation and sections were counterstained with haematoxylin.

**H3K27me3 IHC** was undertaken using a Leica Bond 3 automated immunostainer (Leica Microsystems, Milton Keynes, UK). Epitope retrieval solution (EDTA-based pH 9 buffer solution, BOND Epitope Retrieval Solution 2) was used for 20 min. The antibodies used included H3K27me3 [Tri-Methyl-Histone H3 (Lys27), rabbit monoclonal antibody (clone C36B11), dilution 1/200; Cell Signaling Technology, Danvers, MA, USA].

The immunoreactivity for H3K27me3 was assessed as ‘positive or negative’. Loss of expression was defined as complete absence of nuclear immunoreactivity in the presence of an unequivocal internal positive control, such as endothelial cells or lymphocytes. Only very occasional scattered positive cells were accepted in this category. As the scoring of the cases with partial loss of expression appears to be unreliable [32], such cases were scored as ‘positive’. The expression of H3K27me3 was also assessed in a total of 82 synovial sarcomas on tissue microarrays and recorded as retained or loss.

**SSTR2 IHC** was performed using Ventana automated staining instruments (Ventana Medical Systems, Tucson, AZ, USA) and the rabbit monoclonal antibody UMB1 (Abcam, Cambridge, UK), dilution 1/3000. Antigen retrieval was performed in pH 6.0 citrate buffer in a microwave oven (three cycles 5 min each at 750 W). The immunostaining was optimised on kidney tissue in the presence and absence of the primary antibody. Controls are shown in supplementary material, Figure S12. The slides were dichotomously scored as being positive or negative based on the extent of staining and intensity. The extent was scored on a continuous scale from 0 to 100%. The intensity was scored as three categories as previously reported [33]: 1: weak positivity not easily seen using the low-power objective; 2: moderate positivity seen on a low-power objective; 3: strong positivity easily visible using low-power objective as seen on the kidney control.

*Fluorescence* in situ *hybridization (FISH) analysis*

FISH was performed using the custom commercially available *SSTR2* FISH probe (SSTR2-20-OR; Empire Genomics, Buffalo, NY, USA) and the centromeric probe 17 (CEP17) (Abbott Molecular, Abbott Park, IL, USA). SSTR2 probes are labelled orange and CEP17 in green. In brief, deparaffinised sections were pretreated with deionised water in a pressure cooker for 5 min and digested with pepsin at 37 °C for 50 min. Subsequently, the tissue sections and SSTR2/CEP17 FISH probe were co-denatured at 72 °C for 10 min and hybridised overnight at 37 °C. Following hybridisation, washing was performed. Slides were then counterstained with 4',6-diamidino-2-phenylindole (DAPI) and mounted with coverslips [34].

Amplification was classified as positive if ≥ 10% of the cells showed (a) an SSTR2/CEP17 ratio greater than 2 and (b) more than 15 copies of SSTR2 per cell. Disomy was defined as two copies of the gene of interest and CEP17 in more than 90% of the cells. Polysomy was defined as more than three copies of the gene of interest and CEP17 in more than 10% of the cells [35].

*Frozen material for WES and Illumina arrays*

Frozen tissue samples were sectioned using a cryostat (Leica CM1860 UV; Leica, Milton Keynes, UK). A minimum tumour percentage of > 60% and necrosis < 20% was required for inclusion. DNA from both tumour and matched blood was extracted using a QIAamp DNA Blood Maxi kit (Qiagen, Hilden, Germany) and quantified using Qubit (Thermo Fisher Scientific, Waltham, MA, USA), and stored at –80 °C.

*Whole-exome or whole-genome sequencing*

One microgram of genomic DNA underwent shearing and end repair, followed by phosphorylation and ligation with barcoded sequencing adaptors. DNA was size-selected for fragments of 200–400 basepairs (bp) in length and sequenced on an Illumina HiSeq 2500 with 75 bp reads. The median depth of tumour whole-exome sequencing (WES) was 145x (range 112–214x) and 80x (range 52–107x) for blood germline samples [36]. Two cases underwent whole-genome sequencing (WGS) as previously reported [15]: this was performed to at least 70x and matching blood to at least 30x, and alterations in the coding region were examined. Sequencing reads were mapped to the human reference genome (GRCh37) using Burrows–Wheeler Aligner [37]. PCR duplicates were removed using Sequencing Alignment/Map (SAM) [37] tools (mean/median number of duplicates was 4.71 and 4.75% for normal and tumour samples, respectively).

The sequenced germline samples were aligned to the hg19 reference genome with BWA-MEM and GATK best practice recommendations were applied [38], followed by germline SNP and indel calling. The potential pathogenicity of missense and nonsense germline mutations was assessed by mining public databases such as NCBI, OMIM, Cosmic, ClinVar, and the Human Genome Mutations Database (HGMD) [39], as well as sources gnomAD and ESP, to assess allele frequency. Four different missense variant pathogenicity *in silico* tools, PolyPhen-2, SIFT, MutationTaster, and AlignGVGD, were used to predict the possible impact of the variant on the protein folding, stability, and function [40]. The assessment of the effects of intronic alterations on RNA splicing was determined using MaxEntScan, NNSplicer, and GeneSplicer.

*Single-nucleotide variant (SNV)/indel analysis*

WES data were analysed for somatic SNVs and indels using The Cancer Genome Project variant calling pipeline [41]. The following algorithms were applied using standard settings, without additional post-processing of aligned BAM files: CaVEMan (1.11.0) [42] for substitutions and Pindel (2.1.0) for indels [43]. The precision of the CaVEMan and Pindel pipelines as calling variants has been well studied. However, following calling, all variants were manually inspected using the Integrative Genomics Viewer^®^ (IGV; Broad Institute, Cambridge, MA, USA). Synonymous variants were discarded and the subsequent analysis focused on non-synonymous mutations. Variants were then filtered based on key genes of interest in MPNST in addition to their potential pathogenicity [4,5]. The potential pathogenicity of missense variants was examined using the *in silico* predictive algorithms SIFT and PolyPhen [40,44]. Mutational burden was calculated using 38 Mb as the estimate of the exome size as in Chalmers *et al* [45], while a whole-genome size of 3234.83 was used for the samples run using WGS.

Driver mutational analysis was conducted using the dNdScv R package [46] of the alterations listed in supplementary material, Table S2.

*Overview of sequencing data from the 37 sequenced MPNST cases*

The 37 sequenced MPNSTs (excluding the two epithelioid cases, supplementary material, Table S5) had a median of 43 somatic coding mutations per tumour (range 10–1345; median mutations/Mb per tumour = 1.08 mutation/Mb, range 0.06–3.61) including single-nucleotide variants (SNVs) and small indels, including both synonymous and non-synonymous variants. Of the total 3255 coding mutations detected in the 37 cases, 1675 were non-synonymous: these included 1501 missense, 109 nonsense, 61 frameshift, and one lost start site (supplementary material, Table S2). *NF1* and *TP53* were the most commonly mutated genes, with 23/37 (62%) cases harbouring one of these genes affected (Figure 1B). Three cases (cases 13, 14, and 32) had mutations/deletions in both *NF1* and *TP53*.

*Mutational signature analysis*

Mutational signature analysis was performed on two cases (cases 155 and 157) that showed > 10 somatic mutations/Mb. Analyses were performed for single base substitutions, doublet base substitutions, and indels. The mutational signatures were extracted using two non-negative matrix factorisation (NMF)-based methods: SigProfiler (elaborated method used to generate signatures in COSMIC) and SignatureAnalyser (Bayesian variant of NMF) [47]. NMF determines the signature profiles (looking at base substitutions C>A, C>G, C>T, T>A, T>C, and T>G), and the proportion of contribution of each signature to the overall cancer genome. The specific mutational signatures identified were grouped to give an indication of the aetiology of the signature in question.

*Copy number and whole-genome doubling analysis*

Copy number analysis was run using ASCAT [48] and GISTIC 2.0 [49], and manually inspected in IGV. GISTIC models ploidy and looks for alterations below/above in order to find amplifications and deletions. Whole-genome doubling was estimated by plotting the ploidy estimations from the ASCAT files against the fraction of LOH (the fraction where the B allele is 0). Samples with a ploidy > 2.5 − 1.25*x*, where *x* is the proportion of the genome that is LOH, were classified as genome-doubled. This corresponds to an LOH-adjusted ploidy above 2.5. Samples with an LOH-adjusted ploidy above 5 (> 5 − 2.5*x*) were classified as twice genome-doubled.

*Processing and analysis of methylation data*

The RNOH methylation dataset and Röhrich *et al* methylation data (Heidelberg [12]) were normalised using the functional normalisation algorithm [50] within the *minfi* R package [51]. Probes that (1) failed a detection *P* value of 0.01; (2) mapped to sex chromosomes; (3) contained an SNP; and (4) probes with a bead count of less than 3 in at least 5% of the samples were removed from the dataset prior to analysis. The total number of tumours in the analyses included the RNOH methylation dataset comprising 102/176 cases and 53 undifferentiated pleomorphic sarcomas [15] (see supplementary material, Table S7); and 158 samples from the Heidelberg study [12] (171 downloaded; 1 failed quality control and 12 lacked the full clinical information) including both benign and malignant nerve sheath tumours. This allowed comparison of the epigenomic features of different high-grade sarcoma subtypes. The RNOH MPNSTs included cases also analysed by WES. tSNE analysis was undertaken on the 30 000 probes with the highest variations across all beta values using the R *t-SNE* package and plotted using the data Ellipse function in the R ‘car’ package [52]. Differential methylated regions (DMRs) were identified using Bumphunter from the *minfi* package through champ.DMP [51,53]. Differential methylated probes (DMPs) were identified using the champ.DMP function in ChAMP [53]. Hierarchical clustering plots were constructed using the ‘pheatmap’ package [54]. The oncoPrint was made using the oncoPrint function in the ‘ComplexHeatmap’ R package [55].

PRC2 target genes were found using the following online tool: <https://www.gsea-msigdb.org/gsea/msigdb/genesets.jsp> and the search words ‘PRC2’, ‘EED’, ‘SUZ12’. Together, this gave us a list of 1350 unique genes, which was used to determine the number of differential methylated probes that targeted PRC2 target genes.

*Processing and analysis of RNA sequencing data*

RNA (200 ng) extracted using a Zymo Direct-Zol RNA purification kit was used to prepare libraries using the NEBNext Poly(A) mRNA Magnetic Isolation Module and the NEBNext Ultra II Directional RNA Library Prep Kit (Illumina, San Diego, CA, USA). Sequencing 50 bp single end, 20 million reads. RNA sequencing data were analysed using the DeSeq2 R package. RNA counts were extracted for SSTR2 and plotted against SSTR2 copy number/ploidy to confirm the high expression of SSTR2 in samples shown to have SSTR2 amplification in the genomic data.
