# Supplementary material for: H3K27me3 expression and methylation status in histological variants of malignant peripheral nerve sheath tumours
Source: J Pathol. 2020 Sep 1;252(2):151–64. doi: 10.1002/path.5507 (PMC8432159; doi:10.1002/path.5507)
Supplement: Supplementary file 2 — Figure S1. Histopathological features of the ten MPNSTs showing no germline or somatic mutation in NF1 Figure S2. Whole‐genome doubling estimation Figure S3. Paraspinal case with loss of H3K27me3 immunoreactivity Figure S4. Hierarchical clustering of RNOH cases Figure S5. MeGrp 2: histopathological features Figure S6. Malignant melanoma cases Figure S7. No correlation between MeGroups or H3K27me3 status and survival Figure S8. H3K27me3 loss is not associated with inferior survival in MPNST Figure S9. Cases with genome doubling have a tendency towards shorter overall survival Figure S10. Representative images of SSTR2 FISH analysis Figure S11. RNA sequencing confirms higher expression of SSTR2 in samples with genomic amplification of SSTR2 Figure S12. SSTR2 immunohistochemistry (IHC) controls Figure S13. Representative images of SSTR2 IHC [file PATH-252-151-s002.docx]

**H3K27me3 expression and methylation status in histological variants of malignant peripheral nerve sheath tumours**

I Lyskjær *et al. J Pathol* DOI: 10.1002/path.5507

**Supplementary Figures S1–S13**

**
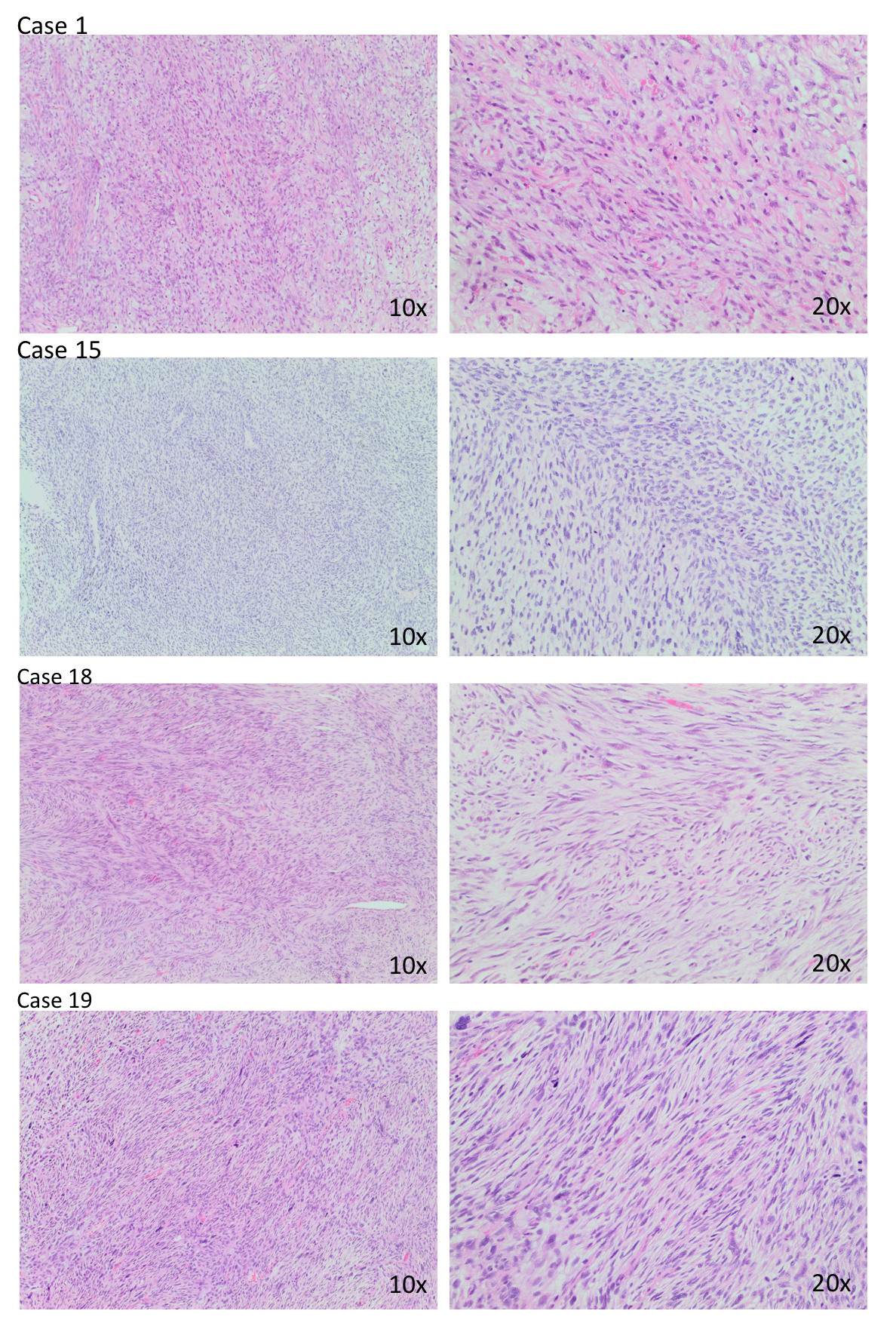
**

**
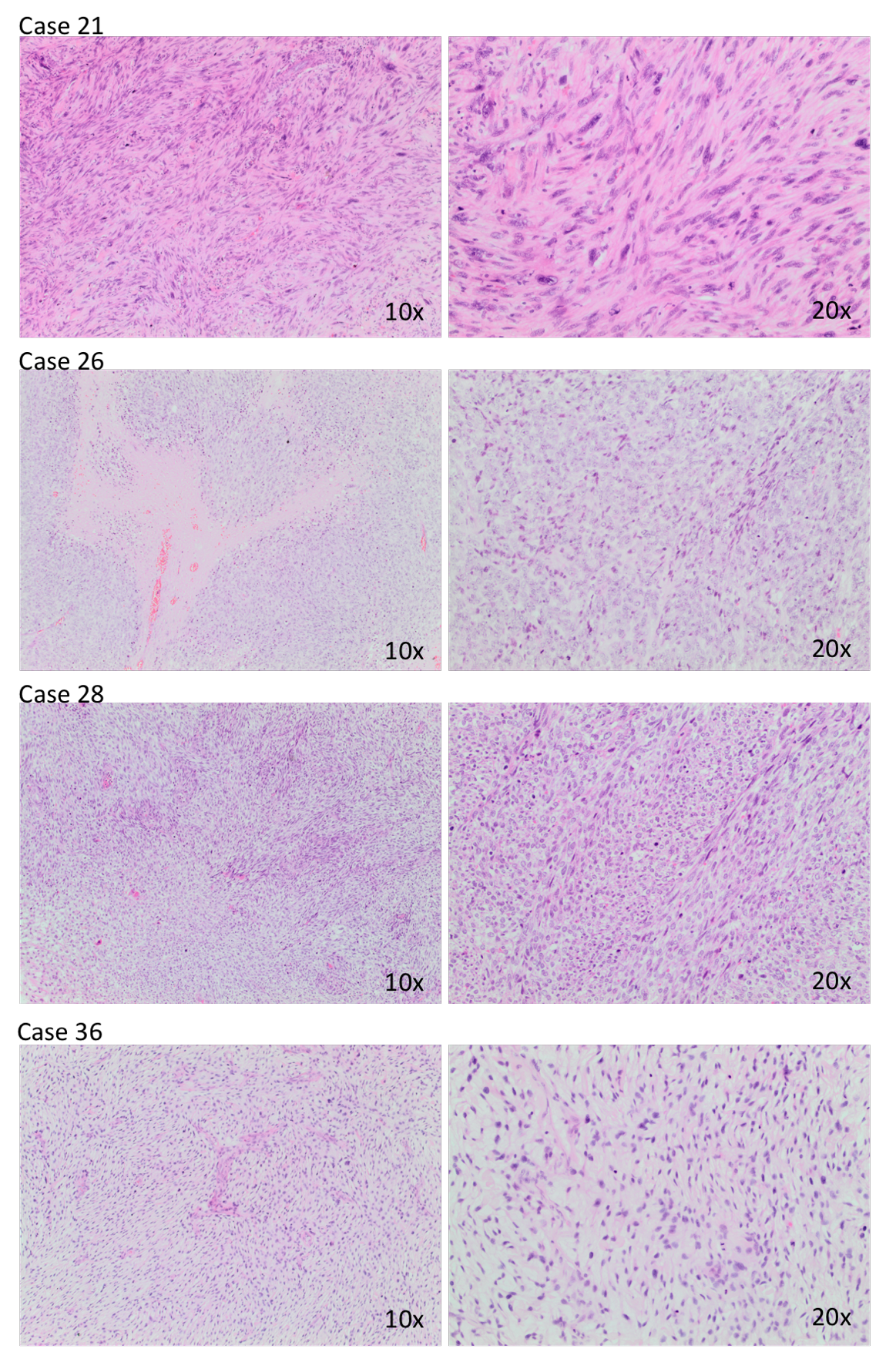
**

**
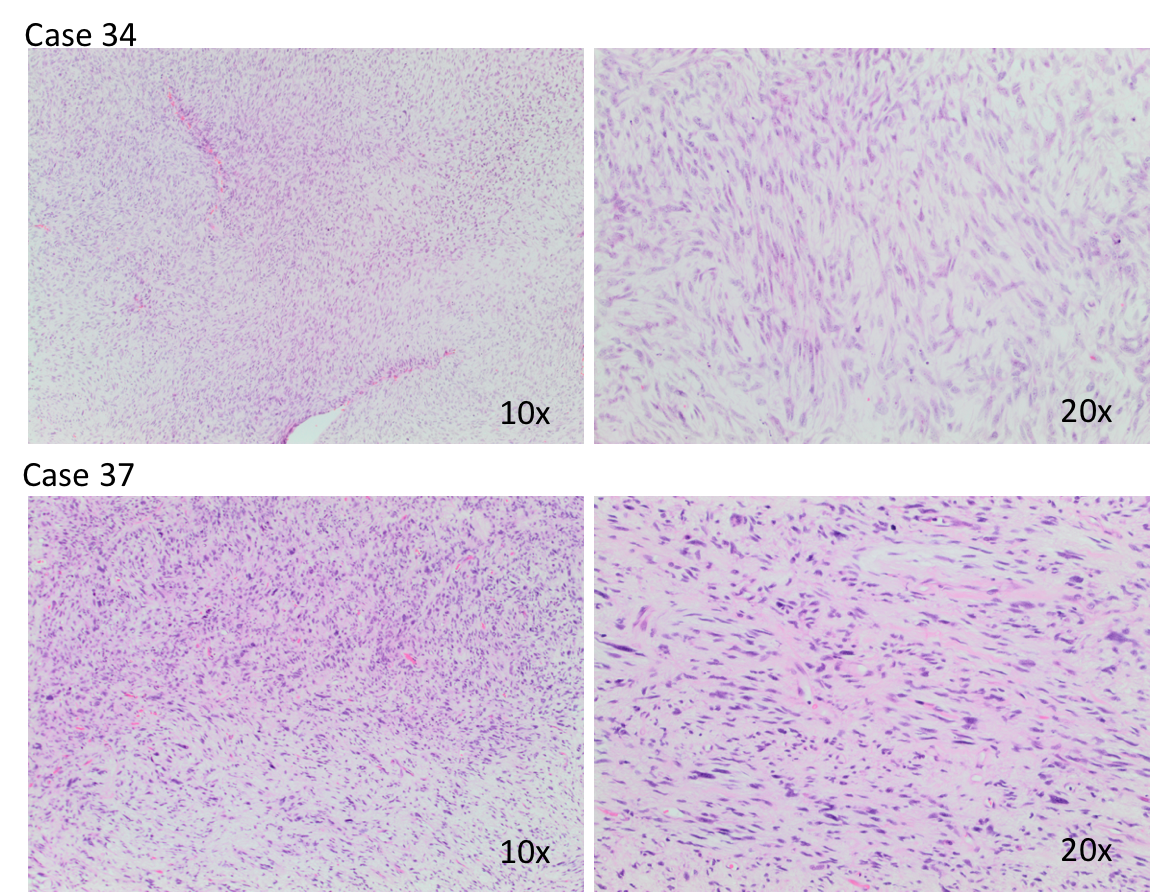
**

**Figure S1. Histopathological features of the ten MPNSTs showing no germline or somatic mutation in *NF1*.**

**Figure S2. Whole-genome doubling estimation.** The proportion of LOH is plotted against the ploidy determined using ASCAT to ascertain whether the 37 MPNSTs are whole-genome-doubled.


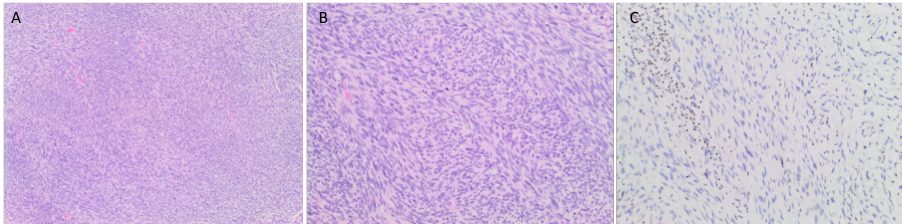


**Figure S3. Paraspinal case with loss of H3K27me3 immunoreactivity.** (A, B) (H&E, ×10; H&E, ×20) Section showing a tumour with a vaguely fasciculated architecture composed of relatively monomorphic elongated spindle cells with dense hyperchromasia, typical of classical MPNST (HP Grp 1A). (C) (IHC, ×10) Section showing complete loss of H3K27me3, with internal positive control (lymphocytes).

**Figure S4. Hierarchical clustering of RNOH cases.** Hierarchical clustering of in-house MPNST and UPS cases using the 10 000 probes with highest variance across all samples. Our in-house methylation dataset comprises 151 cases, including 70 MPNSTs (containing the 37 cases which had undergone DNA sequencing), 54 undifferentiated pleomorphic sarcomas (UPS) (53 from Steele *et al* [15]), four epithelioid MPNSTs, four spindle cell rhabdomyosarcomas (SC-RMS), and 19 sarcomas, NOS. The anatomical site indicates whether the case sample is paraspinal or other. Case numbers are indicated at the bottom of the heatmap. H3K27me3 loss or retained was determined using immunohistochemistry. Note the separation of MPNSTs showing loss of H3K27me3 versus those that have an intact H3K27me3.


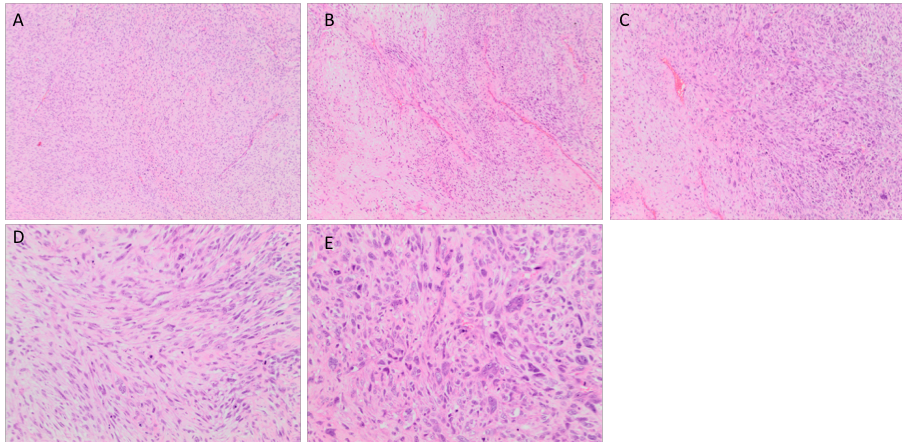


**Figure S5. MeGrp 2: histopathological features.** (A–D) (H&E, ×10; H&E, ×10; H&E, ×10; H&E, ×20) These tumours all showed the characteristic morphology of an SC-RMS, being composed of relatively monomorphic spindle cells with eosinophilic cytoplasm, embedded in a densely collagenised stroma. Desmin and myogenin were positive. All cases exhibited a somatic p.L122R missense mutation in *MYOD1*. (E) (H&E, ×20) This tumour clustered in MeGrp 2 (SC-RMS group). On review, the tumour did not have the typical morphological features of sclerosing rhabdomyosarcoma, instead being composed of more pleomorphic spindled-to-epithelioid cells with a sheet-like architecture. The tumour was also negative for desmin and myogenin.

**Figure S6. Malignant melanoma cases.** t-SNE plot showing clear separation of malignant melanoma from MPNST, epithelioid MPNST, SC-RMS, and UPS. The two cases highlighted in blue (arrow) were originally diagnosed as MPNST on the basis of morphology and negativity for melanocytic markers and were later reclassified following the identification of UV-light-associated mutational signatures.

**Figure S7. No correlation between MeGroups or H3K27me3 status and survival.** (A) Kaplan–Meier plot showing that there was no difference in survival between MPNST cases from MeGroups 4 and 5. (B) Kaplan–Meier plot showing that there was no difference in survival between MeGroup 4 and MeGroup 5 MPNST cases with or without expression of the H3K27me3 protein.

**Figure S8. H3K27me3 loss is not associated with inferior survival in MPNST.** Kaplan–Meier plot showing that there was no difference in survival of patients with high-grade MPNST with loss or retained H3K27me3 (*n* = 100).

**Figure S9. Cases with genome doubling have a tendency towards shorter overall survival.** Kaplan–Meier plot showing that genome doubling (GD) did not correlate with survival in the MPNSTs that were sequenced (*n* = 37).


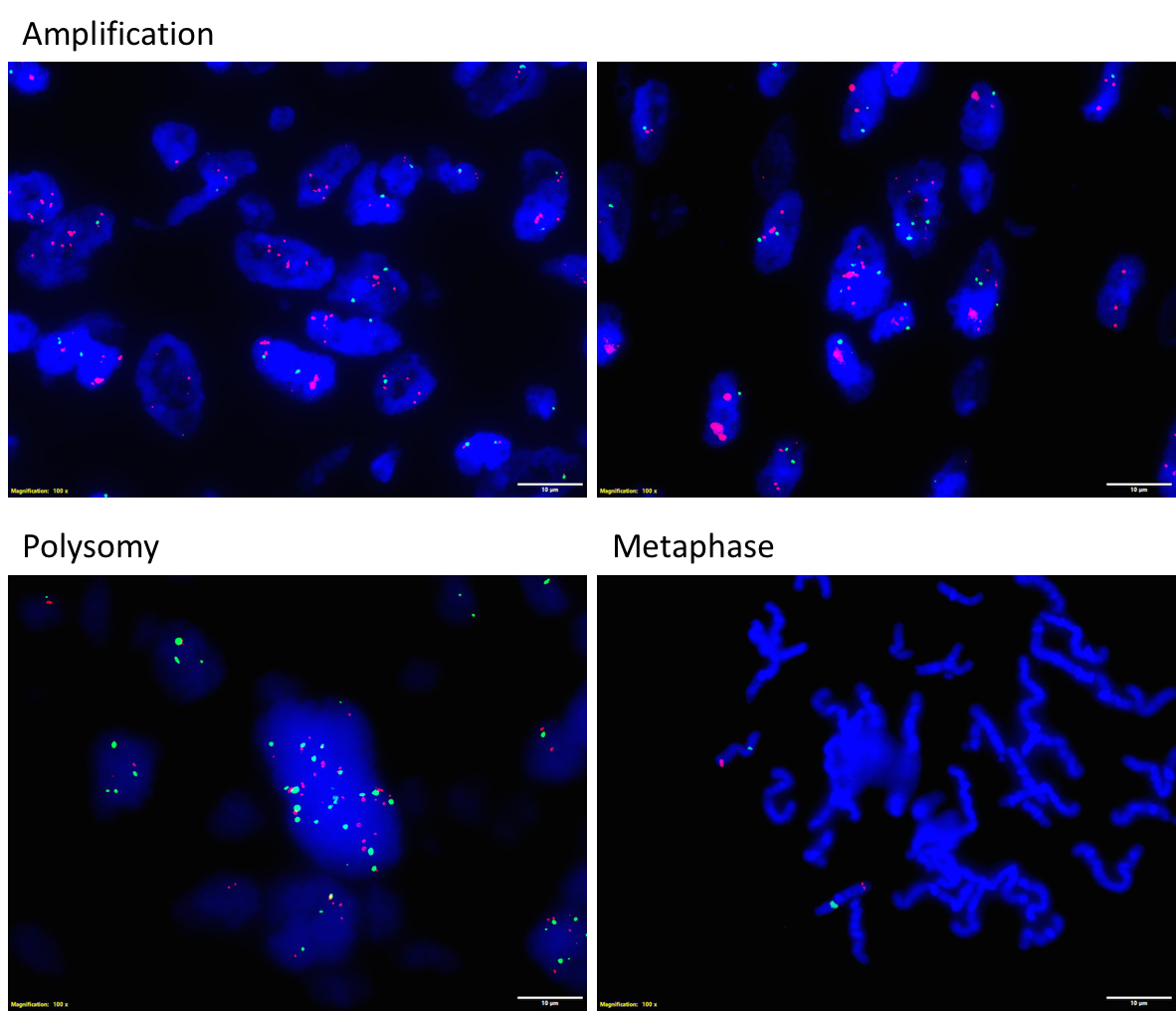


**Figure S10. Representative images of *SSTR2* FISH analysis.** Amplification is shown for cases 27 and 39, while polysomy is shown for case 29.

A:


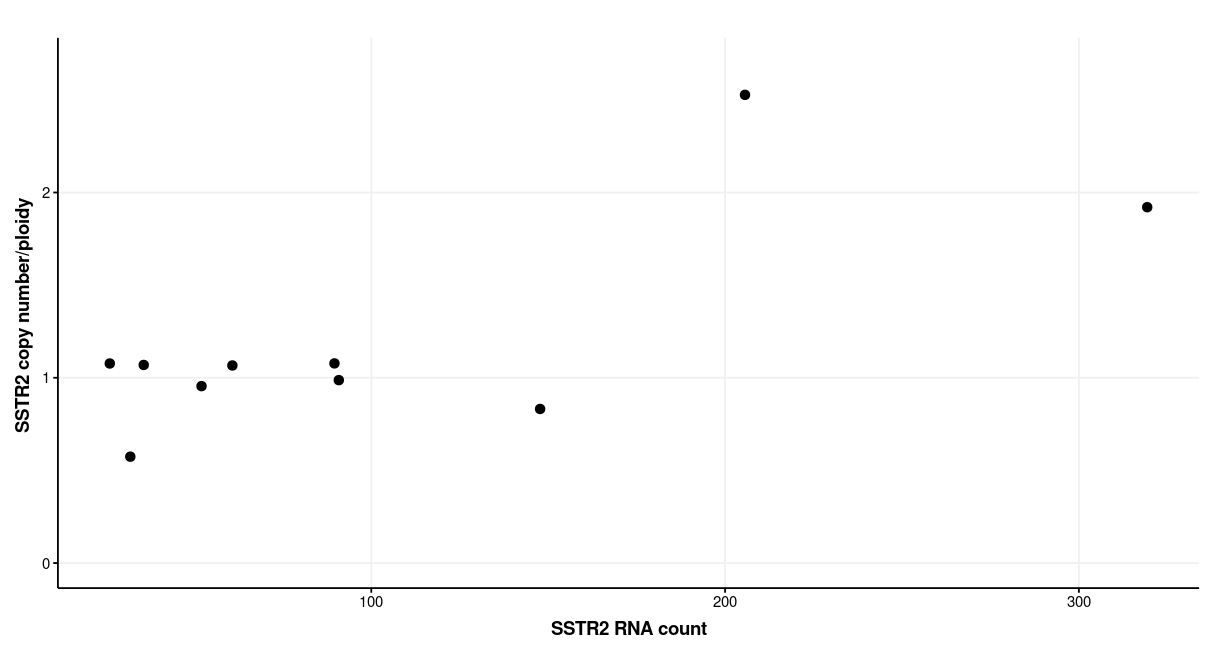


B:


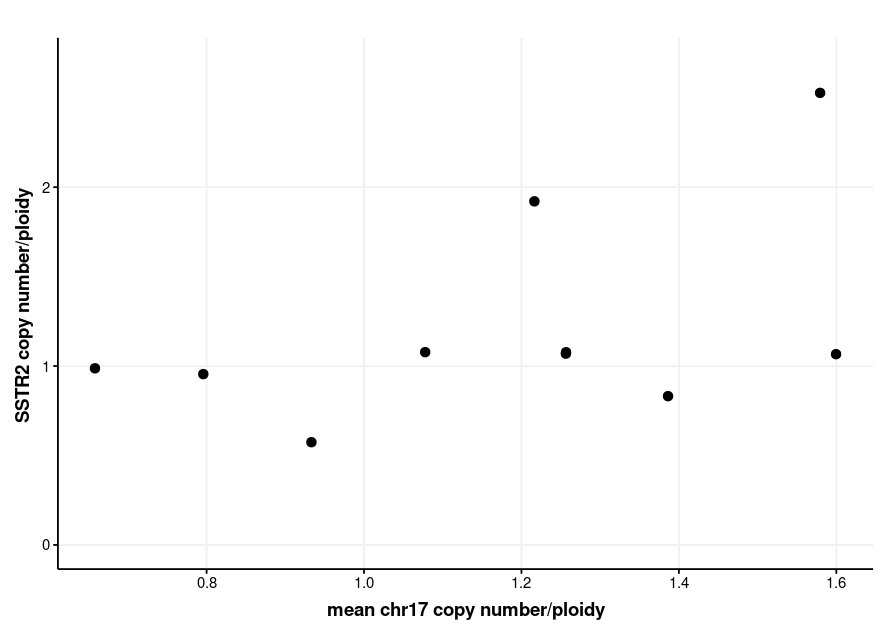


**Figure S11.** **RNA sequencing confirms higher expression of *SSTR2* in samples with genomic amplification of *SSTR2*.** (A) *SSTR2* RNA counts versus *SSTR2* copy number/ploidy determined from ASCAT analysis shows that the two amplified cases have higher expression of *SSTR2* compared with the non-amplified cases. (B) *SSTR2* copy number/mean chromosome (chr)17 copy number versus SSTR2 copy number/ploidy.

**
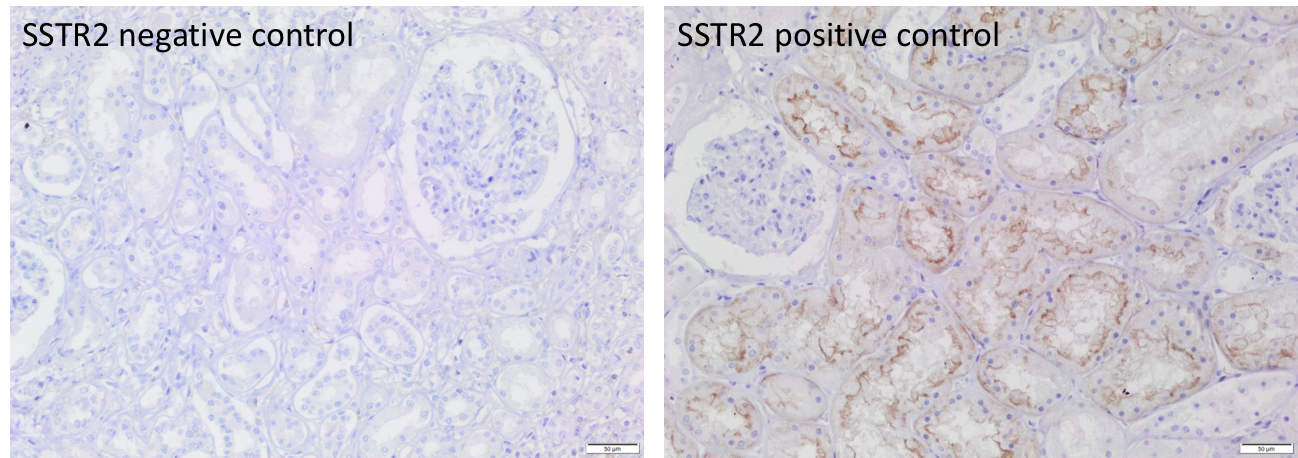
**

**Figure S12. *SSTR2* immunohistochemistry (IHC) controls.**


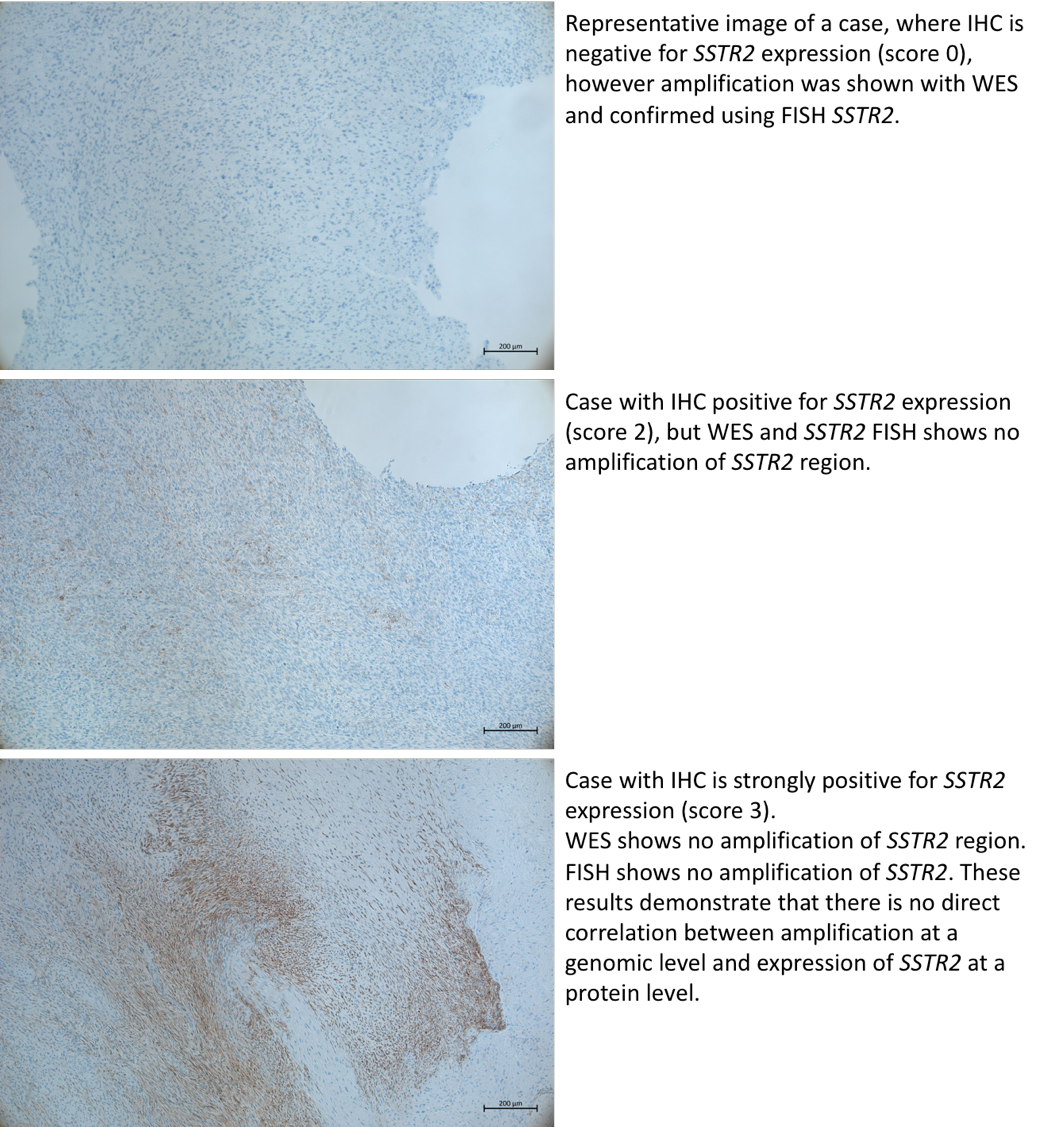


**Figure S13. Representative images of SSTR2 IHC.** 10× objective magnification.
